# Mycotoxin Occurrence in Milk and Durum Wheat Samples from Tunisia Using Dispersive Liquid–Liquid Microextraction and Liquid Chromatography with Fluorescence Detection

**DOI:** 10.3390/toxins15110633

**Published:** 2023-10-29

**Authors:** Khouloud Ben Hassouna, Ahmed M. Hamed, Jalila Ben Salah-Abbès, Kamel Chaieb, Samir Abbès, Ana M. García-Campaña, Laura Gámiz-Gracia

**Affiliations:** 1 Laboratory of Genetic, Biodiversity and Bio-Resources Valorisation, High Institute of Biotechnology of Monastir, University of Monastir, Monastir 5000, Tunisia; khouloudhsn20@gmail.com (K.B.H.); abb_samir@yahoo.fr (S.A.); 2 Laboratory of Analysis, Treatment and Valorization of Environmental Pollutants and Products, Faculty of Pharmacy, University of Monastir, Monastir 5000, Tunisia; chaieb_mo@yahoo.fr; 3 Dairy Science Department, Faculty of Agriculture, Cairo University, Giza 12613, Egypt; ahmed.hamed@agr.cu.edu.eg; 4 Department of Analytical Chemistry, Faculty of Sciences, University of Granada, 18071 Granada, Spain

**Keywords:** mycotoxins, cereals, milk, risk assessment, Tunisia

## Abstract

Food and feed contamination with mycotoxins is a major public health concern. Humans and animals are exposed to these toxins by consuming contaminated products throughout their lives. In this study, a method based on dispersive liquid–liquid microextraction (DLLME), followed by liquid chromatography with fluorescence detection (LC-FLD), was validated for the determination of aflatoxins (AFs) M1, B1, B2, G1, G2, zearalenone (ZEN), and ochratoxin A (OTA). The method was applied to 150 raw cow milk samples and 90 market durum wheat samples from two Tunisian climatic regions: the littoral region (Mahdia) and the continental region (Béja). This work was carried out to obtain more surveillance data to support rapid initiatives to assure safe foods and protect consumer health and to estimate the daily exposure of the Tunisian population consuming those products. AFG2 and OTA were found in wheat with incidences of 54.4 and 11.1%, respectively. On the other side, milk samples were contaminated by AFG2, AFB1, and AFB2 with incidences of 8.7%, 2.0%, and 0.67%, respectively. Some of the samples showed OTA concentrations above the maximum limit allowed by the European Union, which represents a health risk for consumers in Tunisia, where no legislation exists about the maximum content of mycotoxins in food.

## 1. Introduction

Mycotoxins are toxic secondary metabolites with a high capacity for bioaccumulation, thermostability, and low molecular weights that are produced by fungi, in particular, *Aspergillus*, *Penicillium*, and *Fusarium* spp. More than 500 mycotoxins have been identified. The toxicities of aflatoxins (AFs), ochratoxin A (OTA), zearalenone (ZEN), fumonisins (FMs), patulin (PAT), and trichothecenes (TRC) such as deoxynivalenol (DON) and T-2 toxin have been the most investigated [1]. They are responsible for health risks for humans and animals such as immunotoxicity, genotoxicity, hepatotoxicity, nephrotoxicity, teratogenicity, cytotoxicity, and neurotoxicity [2]. Moreover, they have been classified as carcinogenic to humans and animals by the International Agency for Research on Cancer (IARC) [3]. Humans can be intoxicated directly by ingesting contaminated foods (cereals, fruits, legumes, dried fruit, coffee beans, beer, etc.) or indirectly by the consumption of by-products from animals (milk, meat, and eggs) that have consumed contaminated feeds [1]. Cereals are one of the most consumed foods, are the base of most animal feeds, and are subject to mycotoxin contamination [4]. Wheat, barley, and maize constitute the Tunisian population’s primary food sources. They represent about 12% of agricultural production [5]. Wheat crops span over 700,000 ha per year and are mostly produced in the Beja region in the Northern part of Tunisia [6]. Moreover, Tunisia has one of the highest values of wheat consumption per capita of the North African countries, approximately 258 kg/capita/year, and Tunisian people consume a lot of wheat-based products such as bread, couscous, pasta, cakes, biscuits, and bessissa [7].

Milk is also subject to mycotoxin contamination, especially aflatoxin M1 (AFM1), a metabolite of aflatoxin B1 (AFB1). Cow milk is the most commonly consumed milk by humans (83%), followed by buffalo milk (13%), goat milk (2%), sheep milk (1%), and camel milk (1%) [8]. The production of cow milk in Tunisia was around 1462 million liters in 2022, and its consumption is in the order of 110 L/inhabitant/year [9]. Despite the high consumption of cereals (especially wheat) and milk in Tunisia, unfortunately, there are no applicable norms concerning cereal and milk contamination by mycotoxins in most African countries, and, when available, they are only used for international trade [10]. Moreover, to our knowledge, there are only a few recent studies concerning the occurrence of mycotoxins in Tunisian milk and cereals [1,11,12,13,14,15]. Seasonal variations as well as harvest and storage practices can influence mycotoxin concentrations in food, but there is scarce information about the occurrence of mycotoxins in cereals during the harvest and storage seasons in Tunisia. Also, no study has compared the occurrence of mycotoxins in cereals and milk from two different Tunisian regions. Added to that, there are a few studies about the exposure and risk assessment related to the intake of mycotoxins from cereals, in particular, from wheat [13,14,15], but there have been no studies on the evaluation of the risks associated with the ingestion of mycotoxins from milk in Tunisia. 

Regarding the determination of mycotoxins, recent reviews have compiled the most important applications and trends for this topic [16,17,18]. Analytical methods based on chromatographic techniques such as Liquid Chromatography (LC) coupled with tandem mass spectrometry (MS/MS) are the most frequently used because of their high selectivity, sensitivity, and accuracy [19,20]. Moreover, as some mycotoxins show natural fluorescence or can be derivatized to obtain fluorescent products, this detection can also be used with LC, being a cost-effective alternative to LC-MS/MS [21]. The sample treatments are usually based on liquid extractions followed by a clean-up step in order to remove interference. In this sense, miniaturized methodologies, such as dispersive liquid–liquid microextraction (DLLME), which has the advantages of reduced consumption of solvents and high preconcentration factors, has been recently applied to mycotoxin analysis [22,23,24].

Due to the above mentioned reasons and in response to the need for good surveillance data to support rapid initiatives to assure safe foods and protect consumers’ health, this work aimed to determine and quantify mycotoxins (AFs, OTA, and ZEN) in durum wheat (stored and freshly harvested samples) and raw cow milk from Tunisian continental and littoral regions (Beja and Mahdia) by DLLME-LC-FLD and to estimate the daily exposure of Tunisians consuming those products.

## 2. Results and Discussion 

### 2.1. Validation of the DLLME-LC-FLD Method for the Studied Samples

The proposed method based on DLLME-LC-FDL is described in Section 4.4 and Section 4.5 and has been characterized for the studied matrices (milk and wheat). In this sense, performance characteristics such as the linear dynamic ranges, limits of detection and quantification, precision, recovery, and selectivity were evaluated for each analyte.

#### 2.1.1. Calibration Curves, Limits of Detection, and Quantification of the DLLME-LC-FLD Method

Standard calibration curves were established using six different concentration levels for each mycotoxin: 0.1, 0.2, 0.5, 1, 10, and 25 µg/L for AFs; 10, 20, 25, 50, 75, and 100 µg/L for ZEN; and 2, 5, 10, 25, 50, and 75 µg/L for OTA. Each level was prepared in triplicate and injected in duplicate. The slopes, intercepts, and coefficients of determination (*R^2^*) were calculated by least-square regression, while the LODs and LOQs were calculated as 3 × S/N and 10 × S/N, respectively (see Table 1).

#### 2.1.2. Precision Study

Repeatability and intermediate precision were determined through the application of the DLLME-LC-FLD method in blank samples spiked at two concentration levels of mycotoxins: 0.05 and 5 µg/kg for AFs; 5 and 10 µg/kg for ZEN; and 1 and 5 µg/kg for OTA. To check the repeatability, five samples were analyzed and injected in triplicate on the same day under the same conditions (that is, intra-day precision). A similar procedure was carried out in the case of intermediate precision: during five consecutive days, one sample per day was prepared and injected in triplicate (inter-day precision). The repeatability and intermediate precision were estimated as the relative standard deviation (%RSD) and were lower than 8.5% in all cases (see Table 2).

#### 2.1.3. Recovery Study

Recovery experiments were carried out in order to demonstrate the efficiency of the sample treatment in fresh cow milk and wheat samples spiked with two different concentration levels of the studied mycotoxins (the same as for the precision study). The ratio of the peak areas of the samples spiked before extraction and the extracts spiked after extraction were used to calculate the recovery. Table 2 shows the average recovery for each mycotoxin and sample (processed in triplicate and injected three times). The recovery values ranged from 83.9 to 104.5 for the milk samples and 90.3 to 98.9 for the wheat samples.

#### 2.1.4. Selectivity

**Figure 1:**
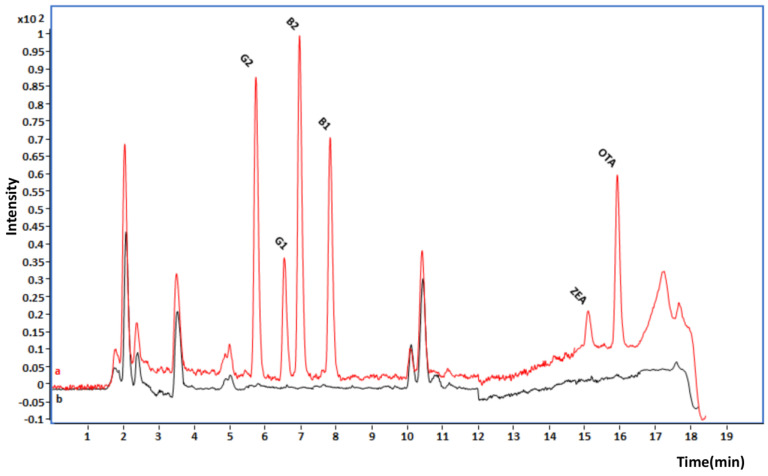
Chromatograms of (a) a spiked wheat sample at 5 µg/kg for AFs, 10 µg/kg for ZEN, and 5 µg/kg for OTA and (b) a blank sample.

**Figure 2:**
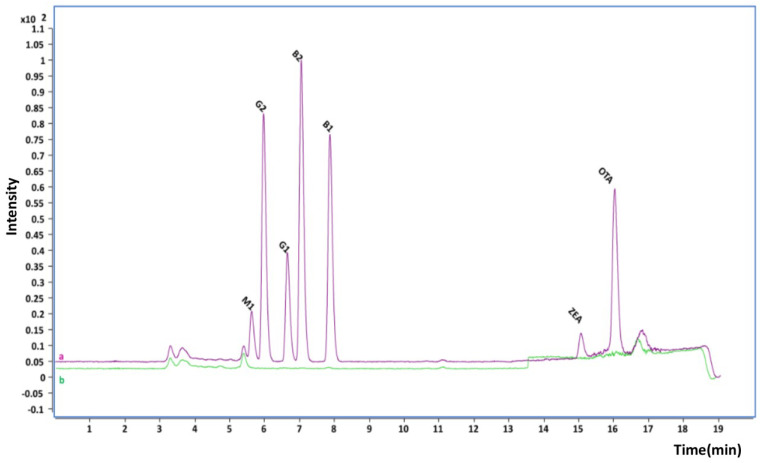
Chromatograms of (a) a spiked milk sample at 5 µg/kg for AFs, 10 µg/kg for ZEN, and 5 µg/kg for OTA and (b) a blank sample.

The selectivity of the method was assessed by comparing the chromatograms of the blank samples and spiked samples. No co-eluting peaks were observed, as can be seen in Figure 1 and Figure 2.

The results obtained for validation fulfill the current recommendations [25].

### 2.2. Occurrence of Mycotoxins in Durum Wheat

A total of 90 samples of wheat were evaluated for the occurrence of AFG1, AFG2, AFB1, AFB2, OTA, and ZEN. Table 3 shows the occurrence, concentration range, and mean concentrations of each mycotoxin in positive durum wheat samples. Samples were considered positive when the concentration was higher than the LOQ.

Concerning the distribution of mycotoxins in positive samples (considering samples with concentrations above the LOQs), 59 out of 90 samples (65.55%) were contaminated by mycotoxins. Moreover, AFG2 was detected in eleven more samples. Among the analyzed mycotoxins, only two were detected, AFG2 and OTA, at concentrations higher than the LOD. 

For example, comparing these results with previously reported studies, in a study conducted by Jedidi et al. [12], the occurrence of aflatoxins AFB1, AFB2, AFG1, AFG2, and OTA was investigated in 65 samples of stored and freshly harvested wheat, barley, and maize collected in Tunisia. The results showed that all contaminated maize samples contained AFG1 and AFG2, with 27.78% of them also having AFB1 and AFB2. In contrast, OTA was not detected in maize or barley samples, and only one wheat sample was found to contain OTA. 

#### 2.2.1. Occurrence of AFG2

AFG2 was the most frequently detected mycotoxin in this study. It was reported in 54.44% (49/90) of the durum wheat samples at concentrations higher than the LOQ (mean concentration of 0.23 µg/kg) and was detected in eleven more samples. The quantified concentrations ranged from 0.12 to 0.58 µg/kg, significantly lower than the maximum limit set by the European Union (EU) for cereals and their derivatives (4 µg/kg for the total AFs content) [26]. Previous studies also reported the presence of AFG2 in cereals from Africa. Thus, Korley et al. [27] detected AFG2 in cereal products from Ghana, with the highest concentration being 0.82 µg/kg. Also, Hathout et al. [28] reported that wheat grain samples collected from Al-Ajami (Alexandria) and Helwan (Cairo) were significantly contaminated by AFG2 with mean values of 8.41 and 7.12 µg/kg, respectively.

Despite the high occurrence of AFG2 in raw wheat, no other AFs were detected. Similar results were shown in Tunisian wheat [29] and in the work of Jedidi et al. [12]. This work suggests the contamination of samples by *A. parasiticus* fungi, and its occurrence in Tunisian cereals was confirmed by the specific PCR assays.

#### 2.2.2. Occurrence of OTA

In our study, OTA was detected only in 11.11% of the analyzed samples (10/90). The OTA concentrations observed were considerably high, ranging from 2.47 to 9.13 µg/kg, with a mean concentration of 5.04 μg/kg. Among the positive samples, 10% had concentrations higher than the EU-authorized maximum content of 5 μg/kg for unprocessed cereals [26]. 

In fact, OTA contamination in wheat or derived products is a global problem [30], in particular, in North Africa [1] and Mediterranean countries. OTA was found in Turkish cereals, including maize (41.7%), wheat (26.7%), and rice (18.8%) [31]. Also, Daou et al. [32] found OTA in Lebanese wheat and products, and Torovic et al. [33] reported that OTA contaminated 20.7% and 13% of Serbian breakfast cereals in 2012 and 2015, respectively. In 2012, 3.6% of the samples showed concentrations higher than the EU maximum allowed level. In another study, OTA occurred in 16.9% of the cereal-based samples (couscous, rechta, and metlou) from Algeria, averaging 0.15 μg/kg, but it was not detected in nuts or dried fruits [34].

In contrast to the low concentrations of OTA detected in our survey, higher concentrations were reported for Algerian wheat grains for the crops from 2012 and 2013, where OTA was detected in 69.23% of samples with quantities ranging from 0.21 to 27.31 μg/kg [35]. The results also showed that 92.85% of the analyzed wheat-derived products were contaminated at levels ranging from 0.25 to 34.75 μg/kg. According to Serrano et al. [36], a higher level of OTA (112 µg/kg) was found in wheat pasta from Tunisia, although OTA was not detected in wheat grain. OTA was also detected in cereals at low concentrations. It was found in two Egyptian wheat grain samples from Al-Shuruq (Cairo) and Esta (El-Fayoum) with mean concentrations of 1.37 and 0.21 μg/kg, respectively, below the maximum concentration (5 μg/kg) set by the Egyptian Organization for Standardization and Quality Control [37]. OTA was also observed in 12% of Lebanese wheat samples at low levels (mean concentration 0.15 µg/kg) [38]. In addition, OTA was found in commercial infant formulas from Burkina Faso with a maximum concentration of 3.2 µg/kg [39].

In a previous study, *Aspergillus niger*, a highly OTA-producing strain, was found to contaminate Tunisian wheat, barley, and maize [12]. The high levels of OTA detected in durum wheat samples may be linked to Tunisia’s Mediterranean climate, which may stimulate the growth of *Aspergillus* and *Penicillium* strains that produce OTA [1]. Furthermore, the contamination of durum wheat samples by mycotoxins may be the result of poor farming practices, such as the use of sensitive cereal varieties, no crop rotation, and no-till or limited tillage. 

Wheat is widely consumed in Tunisia by humans and animals. The high contamination of wheat contributes to the increase in the Tunisian population’s exposure to OTA. Indeed, Tunisian studies have reported significant exposure of the Tunisian population to OTA, and this nephrotoxic mycotoxin (OTA) was detected in their blood at high concentrations [40]. Thus, the presence of mycotoxins in cereals, in particular AFG2 and OTA, even if they appear to be infrequent, necessitates constant monitoring and control.

#### 2.2.3. Distribution of Mycotoxins in Durum Wheat over the Harvest and Storage Seasons

Seasonal variations were noted in mycotoxin concentrations in wheat consumed by the Tunisian people over the harvest and storage seasons (see Table 4), as inadequate storage conditions can result in higher mycotoxin contamination of cereals.

AFG2 concentrations in freshly harvested samples were slightly higher than those that were stored and harvested with levels ranging from 0.12 to 0.58 µg/kg and from 0.12 to 0.43 µg/kg, respectively (mean values of 0.28 µg/kg and 0.41 µg/kg, respectively). In general, it has been reported that AF contamination in cereals has increased due to poor storage practices [41]. Generally, Tunisian people store their cereals in non-hermetic bags (jute and woven polypropylene) that allow air to pass through, exposing the stored foods to fungal spores [42]. Also, differences in climatic factors (temperature and humidity) from one year to another might cause changes in the mycoflora composition of cereals reaching harvest as well as the conditions during posterior storage. Oueslati et al. [29] indicated that AFG2 was the most often found mycotoxin in stored wheat from Tunisia (12%), and other AFs were not detected. However, Jedidi et al. [12] investigated Tunisian maize samples during the storage and harvest seasons and, similar to our findings, they detected low levels (0.08 µg/kg) of AFG2 in stored samples and a higher concentration (18.20 µg/kg) in freshly harvested samples. 

The presence of OTA in cereal grains is determined by the grain status at harvest, the attention with which it is dried, and the storage conditions. OTA, which was classified as a storage mycotoxin, was found in nine stored samples at concentrations ranging from 2.47 to 5.60 µg/kg, with four samples exceeding the maximum limit established by the EU (5 µg/kg). Notably, only one freshly harvested sample of wheat (2–3 weeks after harvesting) was contaminated with OTA, but at a concentration of 9.12 µg/kg, higher than the EU limit. In another study, it was reported that OTA was detected in 30 and 20% of wheat and oat bran samples from Spain, respectively, with mean levels of 1.1 (wheat) and 0.3 (oat) μg/kg. Samples subjected to heat treatment during processing presented a significantly lower concentration of OTA, and also, higher levels of OTA were found in organic samples in comparison with conventionally produced samples [43]. Jedidi et al. [12] detected OTA in only one wheat sample from the stored season at a concentration lower than the maximum value authorized by EU legislation. However, in the study by Oueslati et al. [29], OTA was not detected in Tunisian stored wheat, barley, and their derived products. Similar to our study, Zaied et al. [40] found OTA in 38% of analyzed Tunisian stocked durum wheat samples collected from markets, with a maximum concentration of 250 µg/kg, much higher than the maximum limit set by the EU. Also, Kuruc et al. [44] detected high levels of OTA in stored durum wheat collected over two years (2011 and 2012) from different regions of the United States, with two samples exceeding the maximum limit of the EU. They found this mycotoxin in 30% of samples collected in 2011, with concentrations ranging from 0.17 to 14.94 µg/kg, and in 3% of samples collected in 2012, with concentrations ranging from 0.43 to 12.41 µg/kg. 

The results obtained in this study are most likely due to the presence of hot spots during the transport of wheat before storage, which promote mycotoxigenic fungi proliferation and toxicogenic potential. Indeed, *Penicillium verrucosum*, a producer of OTA, grows best at 25 °C [45], which is the temperature of Tunisia’s wheat culture in June. 

To our knowledge, this is the first comprehensive study on multiple mycotoxin comparisons in durum wheat during the harvest and storage seasons in Tunisia. 

#### 2.2.4. Distribution of Mycotoxins in Durum Wheat in Two Tunisian Regions

The incidences of major mycotoxins in durum wheat grains from the Tunisian littoral and continental regions are presented in Table 5. The results show that 29 out of 38 samples (76.32%) from the littoral region were contaminated by AFG2. On the other hand, 30 out of 52 samples (57.69%) from the continental region were contaminated by AFG2 or OTA.

Moreover, only one sample from the continental region was contaminated by OTA with a concentration of 9.13 µg/kg, which surpasses the maximum limit of the EU (5 µg/kg). Our findings are in accordance with the results of Lahouar et al. [46], who detected OTA in Tunisian sorghum from the Sahel region (littoral region) with concentrations ranging from 1.04 to 27.8 µg/kg and a mean value of 1.71 ± 0.76 µg/kg. Moreover, Lasram et al. [47] did not find OTA in pearl millet collected from the Tunisian littoral region of Mahdia. Furthermore, there were no significant differences in concentrations reported during the two years of sampling for two regions.

As stated before, in our study, only the wheat samples collected from the continental region were contaminated by OTA. This could be due to the impacts of the location, weather, fungal sporulation, cultural practices, and cultivar properties on OTA accumulation in this region. In fact, wheat grain harvested at high humidity levels (>16%) may be infected with field molds, such as *Aspergillus* and *Penicillium*, potentially contaminating it with mycotoxins [45]. Table 6 and Table 7 show that the continental region had higher humidity percentage than the littoral zone during both periods of wheat sampling.

In previous work, cereals from the littoral region were found to be contaminated with other mycotoxins that are not reported in our study. Lahouar et al. [46] reported the contamination of Tunisian sorghum collected from the Sahel region by AFB1 and ZEN with mean concentrations of 1.24 ± 0.34 and 8.56 ± 2.15 µg/kg, respectively. Also, barley collected from Enfidha (Sousse) was contaminated by ZEN at levels ranging from 0 to 52 µg/kg with an average of 47 µg/kg. The variation in the mycotoxin distribution between regions and the variations in their reported concentrations in this comparative study may be due to differences in the climate and environmental conditions (temperature and humidity) from humid to arid regions in the field throughout the growing season (Table 6 and Table 7), which influence the distribution of infecting fungal species and lead to geographical variation in the species distribution [48,49]. 

#### 2.2.5. Co-Occurrence of Mycotoxins in Durum Wheat

Nine samples, all of them from the continental region (that is, an incidence of 17.30% of the samples from this region), were contaminated with the two mycotoxins detected (AFG2 and OTA).

The co-occurrence of mycotoxins in cereals and cereal products has been studied previously in other Mediterranean regions. It was found that a high percentage (51%) of Moroccan wheat grain samples were contaminated with two to six mycotoxins [50]. Also, a high percentage (65%) of cereal-derived samples from Spain presented co-occurrences of several mycotoxins [51]. Furthermore, the co-occurrence of three mycotoxins together (DON + ENB + ENB1) was found in 65% of cereal biscuits from Tunisia [13].

In our findings, although mycotoxins were present in low concentrations in the analyzed samples, the co-occurrence of both nephrotoxic OTA and genotoxic–carcinogenic AFG2 may contribute to synergic effects, thus increasing the health risk caused by these toxins when consumed by animals and/or humans [52]. For these reasons, it may be necessary to confirm the potential effects of their synergistic activity, revise the regulation of their limits in cereals, or establish legislative values for unregulated mycotoxins. In addition, it is essential to detoxify cereal grains before human consumption to decrease mycotoxin concentrations and minimize health risks.

### 2.3. Occurrence of Mycotoxins in Raw Cow Milk

The analysis showed that 17 out of 150 (11.3%) raw cow milk samples were contaminated with different AFs (concentrations higher than the LOQ), while OTA and ZEN were not detected (see Table 8). AFG2, AFB2, and AFB1 were found with incidences of 8.67, 2, and 0.67%, respectively. One sample was contaminated with AFB2 at a concentration lower than the LOQ. In addition to the highest incidence, the highest concentration found was also for AFG2 (up to 0.72 µg/kg). AFB1 was detected only in one sample at a concentration of 0.38 µg/kg. 

The most common mycotoxin evaluated in milk is AFB1, because it is the only mycotoxin with a maximum level established by the different regulations in this matrix. AFM1 is the hydroxylated metabolite of AFB1. AFM1 is found in the milk of animals fed on foodstuffs contaminated with AFB1. Thus, the presence of AFM1 in milk is determined by the concentrations of AFB1 absorbed by the animal. It is estimated that approximately 5% of digested AFB1 is converted into AFM1 in the liver and excreted in the milk of dairy animals [52]. As stated before, AFM1 was not detected in any of the analyzed samples. However, different studies show the incidence of AFM1 in milk and dairy products in the Mediterranean area [53,54,55], and Akinyemi et al. [53] detected AFB1 and AFB2 in fourteen out of one hundred and thirty-five and in one out of one hundred and thirty-five Nigerian milk samples from goat, camel, and cow milk, respectively.

Milk is a natural nutritional food for humans, particularly for children. Even a low level of AF exposure has been linked to chronic risks for humans, such as hepatocellular carcinoma, suppressed immunity, and childhood stunting [56]. The EU fixed 50 ng/L as a maximum concentration authorized for AFM1 in milk destined for the consumption of adults in all circumstances [26], and there are no regulations in Tunisia regarding mycotoxins in milk. Thus, the presence of AFs in Tunisian milk should be controlled from the production step to consumption, as well as by adopting appropriate mycotoxin detoxification processes.

#### 2.3.1. Distribution of Mycotoxins in Raw Cow Milk in Two Tunisian Regions

Table 9 shows the frequency, ranges, and average concentrations of mycotoxins detected in raw cow milk from two different regions (continental and littoral).

The results show that five out of seventy-five milk samples from the littoral region were positive for mycotoxins. On the other hand, 12 out of 75 milk samples from the continental region were contaminated by mycotoxins. AFB2 and AFG2 were present in samples from the two regions investigated (considering only concentrations above LOQs). The mean concentrations of contaminated samples in the littoral region were 0.31 and 0.44 µg/kg for AFB2 and AFG2, respectively. On the other hand, the mean values in the continental region for AFB2 and AFG2 were 0.30 and 0.38 µg/kg, respectively. So, there was no substantial difference between the two regions.

Concerning AFB1, it was present in only one milk sample from the continental region at a concentration of 0.38 µg/kg. 

In the literature, to the best of our knowledge, there is only one study available about the natural incidence of mycotoxins in Tunisian milk and milk products, where 112 raw cow milk samples from the continental region (Beja) were analyzed [11], including only AFB1 and AFM1. Contrary to our results, they detected only AFM1 in 60.7% of the samples with a mean concentration of 13.62 µg/L. However, this study is from 2012, so the results are not comparable with the current scenario.

Our study reported that the continental region presented a higher percentage of contaminated samples than the littoral region (16% vs. 6.66%). The variation in milk contamination between the continental and littoral regions may be due to different factors such as the animal species, the sampling month collection (April and May for the continental and littoral regions, respectively), the milking time, the level of mycotoxins present in the feed intake, and the volume of milk produced by the animal mammal.

#### 2.3.2. Co-Occurrence of Mycotoxins in Raw Cow Milk

In this survey, the co-occurrence of mycotoxins in raw cow milk was reported. The results show that 4% of the positive samples were contaminated with two mycotoxins (AFG2 and AFB2). Moreover, one sample (1.33%) was contaminated by the three mycotoxins detected (AFG2, AFB1, and AFB2).

The co-occurrence of these mycotoxins in foods reflects a serious public health problem, which is highly associated with human aflatoxicosis, neural tube defects, and many types of cancer. In addition, the presence of aflatoxins in milk may cause growth, development, and performance problems in lactating animals [57].

### 2.4. Risk Assessment

The Tunisian population (considering a body weight of 70 kg) consumption data used in this study for wheat and milk were 716.66 g/day and 0.30 L/day [9,58], respectively. The results allowed us to evaluate the risk assessment related to the intake of mycotoxin in wheat by estimating the probable daily intake (PDI) for OTA. According to the EFSA [59], the tolerable daily intake (*TDI*) for OTA is 17 ng/kg bw/day.

No *TDI* was established for AFs, and it is assumed that the presence of AFs in foods should be kept to a minimum (“as low as reasonably achievable: ALARA”), because they are carcinogenic compounds.

The results obtained from this work are summarized in Table 10. As shown, the mean Estimated Daily Intake (*EDI*) for OTA from wheat grain samples obtained from Tunisia was 51.6 ng/kg b.w./day. The Hazard Index (*HI*) value for OTA in wheat samples in this study was 3.03. 

The *EDI* represents more than three times the *TDI* for OTA (303.2%), corresponding to an *HI* (3.03) of much higher than 1, indicating a health risk for consumers.

## 3. Conclusions

In this work, DLLME-HPLC-FD was used to detect multi-mycotoxins in raw cow milk and durum wheat from Tunisia. AFG2 (incidence of 54.4% in wheat and 8.7% in milk) and OTA (incidence of 11.1% in wheat) were the most detected mycotoxins, while ZEN, AFG1, and AFM1 were not detected, and AFB1 was found in just one milk sample. AFG2 and OTA levels were found to be high in milk and wheat samples, respectively. In fact, only wheat from the continental region was contaminated by OTA, which is attributable to differences in climate conditions (particularly humidity percentages and the water activity (Aw)) between the two regions.

From a toxicological standpoint, the determined exposure criteria (*EDI* and *HI*) indicate that the Tunisian population is exposed to OTA through the consumption of durum wheat, which can result in health risks for those consumers. Thus, it is necessary to carry out adequate agricultural practices to reduce mycotoxins in Tunisian wheat and milk.

To protect human health, strict hygienic conditions for milk production, changes in cereal storage habits, more research on the occurrence of mycotoxins in foods, and the sensitization of farmers to the negative effects of mycotoxins should all be considered. Furthermore, regulations on mycotoxins in milk and cereals should be established in Tunisia, and various methods for mycotoxin prevention and detoxification should be considered.

## 4. Materials and Methods

### 4.1. Chemicals and Reagents

All reagents were of analytical reagent grade unless otherwise indicated, and solvents were of LC-MS grade. Ultrapure water was obtained from a Milli-Q Plus system (Millipore Bedford, MA, USA). Methanol (MeOH) and acetonitrile (MeCN) were purchased from VWR International Eurolab (Barcelona, Spain), formic acid was purchased from Sigma Aldrich (St. Louis, MO, USA), and NaCl was purchased from Panreac Química (Barcelona, Spain). Analytical standards for each AF were supplied by Sigma-Aldrich (St. Louis, MO, USA). Individual stock standard solutions containing 1 μg/mL of each compound were prepared by dissolving accurately weighed amounts in MeCN. These solutions were stable for at least 6 months. Standard solutions (10 mg/L in MeCN) of OTA and ZEN were purchased from Techno Spec (Barcelona, Spain). From these stock solutions, multi-mycotoxin intermediate working solutions in MeCN:MeOH:water (25:25:50 *v*/*v*) were prepared by combining suitable aliquots of each individual standard stock solution. All of these solutions were kept at −20 °C. Nylon syringe filters (13 mm, 0.22 μm from VWR) were used for the filtration of extracts before injection into the chromatograp c system.

### 4.2. Instruments and Equipment

Mycotoxin determinations were carried out using an Agilent 1290 Infinity LC system (Agilent Technologies, Waldbronn, Germany) with an Infinity LabPoroshell 120 EC-C18 separation column (100 × 4.6 mm, 2.7 μm). In order to enhance the fluorescence of AFB1 and AFG1, a photochemical derivatization module (LCTech GmbH, Obertaufkirchen, Germany), which consisted of a 254 nm lamp, was placed between the column and the fluorescence detector (Agilent 1260 FLD Spectra).

For sample treatment, a high-speed solids crusher with a grinding degree of 50–300 mesh and a rotation speed of 25,000 rpm (Model 250A from Hukoer, China), a vortex-2 Genie (Scientic Industries, Bohemia, NY, USA), an evaporator system (SystemEVA-EC, from VLM GmbH, Bielefeld, Germany), and a universal 320R centrifuge (Hettich Zentrifugen, Tuttlingen, Germany) were used.

### 4.3. Samples

All samples of raw cow milk (*n* = 150) and wheat (*n* = 90) destined for human consumption were collected in 2020–2021 from two different geographical regions and climatic conditions in Tunisia: Beja, a continental region that presents a higher-arid climate, and Mahdia, a littoral region characterized by a higher semi-arid climate. Table 11 summarizes the geographic characteristics of the littoral and continental regions.

Concerning wheat, 33 samples (fresh samples) were collected at harvest time from fields, and 57 (stored samples) were randomly collected from cereal retailers (shops, markets, small groceries, and specialized cereal stores) situated in Beja and Mahdia (Tunisia). Sampling was conducted in two periods: the first period was after six months of the harvest of 2020 (post-storage samples), and the second period corresponded to 2–3 weeks after the harvest of 2021 (post-harvest samples). Samples were then weighed (100 g each sample), packed in polyethylene bags, and stored at −20 °C until analysis.

For the milk, all fresh samples (50 mL each) were collected from cows during a routine midday milking procedure. Samples were collected from farms located in Beja and Mahdia during the spring season of 2021 and were stored in 50 mL falcon tubes at −20 °C until analysis, without being treated in any way beforehand.

All samples were transported to Granada (Spain) in polystyrene containers containing dry ice and kept at −20 °C until analysis. Table 12 presents the sampling details.

### 4.4. Sample Preparation

The sample treatment was based on a previous study on the determination of AFs in slightly modified dairy products [22]. Briefly, milk samples were defatted by centrifugation (6000 rpm, 4 °C, 5 min). A 5 g portion of a defatted sample was placed in a 15 mL falcon tube, and 1.5 g of NaCl and 6 mL of MeCN were added to the sample. The mixture was shaken by vortex for 30 s and centrifuged (6000 rpm, 5 min). Subsequently, the upper phase was quantitatively transferred (4  ±  0.2 mL) into a 10 mL vial. A mixture of this organic phase containing the extracted mycotoxins (disperser solvent) and 1000 μL of CHCl_3_ (extractant solvent) was rapidly injected into 5 mL of deionized water for DLLME. Then, this solution was strongly shaken (30 s), the mixture was centrifuged (5 min, 6000 rpm), and the resulting organic phase was collected and dried under a nitrogen stream. The final residue was dissolved in 500 μL of MeCN:MeOH: water (25:25:50 *v*/*v*) and filtered before the HPLC analysis.

On the other hand, a previous solid–liquid extraction was necessary for wheat samples, which were prepared as follows: a 2 g portion of sample was placed in a 50 mL falcon tube. Then, 10 mL of MeCN (5% formic acid) was added to the sample. The mixture was shaken for 30 s by vortex and centrifuged for 5 min at 6000 rpm. Subsequently, 5 mL of the upper phase was quantitatively transferred into a 10 mL vial. A mixture of this organic phase containing the extracted analytes (disperser solvent) and 2000 μL of CHCl_3_ (extractant) was injected into 5 mL of deionized water for DLLME. After that, the procedure continued as for the milk samples.

### 4.5. Chromatographic Separation and Detection

The chromatographic separation was performed on an InfinityLab Poroshell 120 EC-C18 separation column (100 × 4.6 mm, 2.7 μm). The mobile phase consisted of a linear gradient of solvent A (water) and solvent B (MeCN:MeOH, 50:50), both acidified with 0.2% acetic acid. The initial gradient condition was 35% B, changing to 43% B in 8 min, then changing in 8 min to 60% B, and finally, returning to the initial conditions and maintaining them for 4 min for column equilibration. The flow rate was set at 1.2 mL/min, the column temperature was 50 °C, and an injection volume of 10 μL was selected. A photochemical reactor with a 254 nm lamp was used to enhance AFB1 and AFG1 fluorescence. The wavelengths of excitation and emission were fixed at 365 and 440 nm for AFs and 234 and 458 nm for ZEN and OTA, respectively. The fluorescence detector operated at gain ×10.

### 4.6. Risk Assesment

#### 4.6.1. Estimation of the Daily Intake

The Estimated Daily Intakes (*EDIs*) of AFG2 and OTA in wheat and AFB1, AFB2, and AFG2 in milk samples were calculated as follows [60]:

(1) $$EDI=\frac{C\times ADC}{BW}$$

where

*C* is the concentration of mycotoxins in wheat or milk (ng/kg);
*ADC* is the average daily consumption of wheat or milk (kg/person/day);
*BW* is the body weight (kg).

#### 4.6.2. Hazard Index (HI)

To calculate the HI value, the estimated intake was divided by the tolerance daily intake value (*TDI*), which was 17 ng/kg b.w./day for OTA. An *HI* of less than one typically indicates a low consumer risk.

### 4.7. Statistical Analysis

The validation of the analytical method was conducted on the basis of the Harmonized Guidelines for Single Laboratory Validation of Methods of Analysis (Thompson, Ellison, & Wood, 2002). This method was initially validated by an analysis of replicates of standard solutions and spiked samples (*n* = 6) at 5.0 ng/g for durum and soft wheat. Spiked samples were used 1 h prior to the extraction for equilibration. Additionally, a matrix blank was analyzed to evaluate any remaining mycotoxin amount. Data were analyzed by the SPSS software (SPSS Institute, Inc., CA, USA, 2000, Version 10.0), and a means comparison was made by the ANOVA test (*p* < 0.05).

## Figures and Tables

**Table 1:** Calibration curves, LODs, and LOQs.

Analyte	Slope	Intercept	*R*^2^ (%)	LOD (µg/L)	LOQ (µg/L)
AFM1	0.156	0.041	99.64	0.014	0.045
AFG2	0.531	0.068	99.91	0.006	0.020
AFG1	0.237	0.046	99.94	0.014	0.046
AFB2	0.728	0.117	99.96	0.004	0.012
AFB1	0.531	0.089	99.93	0.005	0.016
ZEN	0.010	0.011	99.28	0.220	0.733
OTA	0.100	0.080	99.14	0.042	0.139

**Table 2:** Precision and recovery studies.

	Analyte	Repeatability (*n* = 15)		Intermediate Precision (*n* = 15)		Recovery (*n* = 9)	
		Level 1 ^a^	Level 2 ^b^	Level 1 ^a^	Level 2 ^b^	Level 1 ^a^	Level 2 ^b^
Milk samples	AFM1	7.7	4.4	7.7	4.3	94.3	95.4
	AFG2	5.7	4.2	7.2	5.3	99.0	104.5
	AFG1	4.8	3.9	8.3	4.7	93.6	102.3
	AFB2	4.1	3.6	6.4	3.8	94.8	100.5
	AFB1	5.8	3.7	8.5	4.6	96.4	100.3
	ZEN	5.1	4.7	8.0	7.1	96.1	97.9
	OTA	4.4	3.4	4.7	3.1	83.9	86.1
Wheat samples	AFG2	3.5	4.7	6.8	5.8	96.6	98.9
	AFG1	4.6	4.7	7.8	6.3	93.7	93.9
	AFB2	2.8	3.1	7.9	6.7	97.9	93.8
	AFB1	4.2	4.1	6.8	5.4	97.3	95.8
	ZEN	4.4	3.5	7.5	6.6	94.0	94.3
	OTA	3.8	4.8	5.9	4.9	90.3	92.9

^a^ 0.05 µg/kg for AFs; 5 µg/kg for ZEN; and 1 µg/kg for OTA. ^b^ 5 µg/kg for AFs; 10 µg/kg for ZEN; and 5 µg/kg for OTA.

**Table 3:** Results of mycotoxin occurrence in durum wheat samples.

Nº of Samples	Nº Positive ^a^/ Total Samples (Incidence, ^b^ %)	Detected Mycotoxins	Nº Positive ^a^/ Total Samples (Incidence, ^b^ %)	LOQ-LOD ^c^	Mean conc. (µg/kg) ^d^	Range (µg/kg)
90	59 (65.55%)	AFG2	49 (54.44%)	11	0.23	0.12–0.58
		OTA	10 (11.11%)	-	5.04	2.47–9.13

^a^ Number of positive samples with concentrations > LOQ; ^b^ incidence of samples with concentrations > LOQ (% of samples > LOQ); ^c^ number of samples with concentrations between the LOD and LOQ; ^d^ mean value for samples with concentrations > LOQ.

**Table 4:** Distribution of mycotoxins detected in durum wheat grains.

Samples	Nº Positive ^a^/ Total Samples (Incidence, ^b^ %)	Detected Mycotoxin	Nº Positive ^a^/ Total Samples (Incidence, ^b^ %)	Mean conc. (µg/kg) ^c^	Range (µg/kg)
Stored harvest (n = 57)	40/57 (70.17%)	AFG2	31 (54.39%)	0.41	0.12–0.43
		OTA	9 (15.79%)	4.59	2.47–5.60
Freshly harvest (n = 33)	19/33 (57.57%)	AFG2	18 (54.55%)	0.28	0.12–0.58
		OTA	1 (3.03%)	9.12	

^a^ Number of positive samples > LOQ; ^b^ incidence of samples > LOQ (% of samples > LOQ); ^c^ mean value for samples > LOQ.

**Table 5:** Incidence of the detected mycotoxins (µg/kg) in durum wheat samples from the two different regions.

Sampling Region	Nº Positive/Total Samples (Incidence, %)	Detected Mycotoxin	Nº Positive ^a^/ Total Samples (Incidence, ^b^ %)		LOD-LOQ ^c^		Mean conc. ^d^ (µg/kg)		Range (µg/kg)		RSD (%)	
			2020	2021	2020	2021	2020	2021	2020	2021	2020	2021
Littoral Region (Mahdia)	29/38 (76.32%)	AFG2	18/27 (66.66)	11/11 (100)	3	0	0.17	0.30	0.11–0.40	0.18–0.58	39.10	37.72
Continental Region (Beja)	30/52 (57.69%)	AFG2	13/30 (43.33)	7/22 (31.81)	2	6	0.23	0.23	0.11–0.43	0.11–0.37	43.15	38.48
		OTA	9/30 30	1/30 3.33	0	0	4.58	9.13	2.47–5.6	9.13	20.29	0

^a^ Number of positive samples > LOQ; ^b^ incidence of samples > LOQ (% of samples > LOQ); ^c^ number of samples < LOD and >LOQ; ^d^ mean value for samples > LOQ.

**Table 6:** Meteorological conditions during the wheat storage period in 2020–2021. Data from the Tunisian Institute of Meteorology (https://www.historique-meteo.net/, accessed on 23 July 2023).

Region and Period	Average Temperature (°C)	Humidity (%)
Littoral region June 2020–March 2021	21.3	70.0
Continental region June 2020–March 2021	19.4	75.2

**Table 7:** Meteorological conditions during the wheat development period of 2021. Data from the Tunisian Institute of Meteorology (https://www.historique-meteo.net/, accessed on 23 July 2023).

Region and Period	Average Temperature (°C)	Humidity (%)
Littoral region March–May	18.3	74.0
Continental region March–May	16.7	79.3

**Table 8:** Mycotoxin occurrence (µg/kg) in raw cow milk samples.

Total Nº of Samples	Nº Positive ^a^/ Total Samples (Incidence, ^b^ %)	Detected Mycotoxins	Nº Positive ^a^/ Total Samples (Incidence, ^b^ %)	LOD-LOQ ^c^	Mean conc. (µg/kg) ^d^	Range (µg/kg)	RSD (%)
150	17 (11.3%)	AFG2	13 (8.67%)	1	0.35	0.03–0.72	57.84
		AFB2	3 (2%)	-	0.30	0.24–0.36	21.20
		AFB1	1 (0.67%)	-	0.38	-	-

^a^ Number of positive samples with concentrations > LOQ; ^b^ incidence of samples with concentrations > LOQ (% of samples > LOQ); ^c^ number of samples with concentrations between the LOD and LOQ; ^d^ mean value for samples with concentrations > LOQ.

**Table 9:** Incidence of the studied mycotoxins (µg/kg) in raw cow milk samples for two different regions.

Sampling Region	Nº Positive/Total Samples (Incidence, %)	Detected Mycotoxin	Nº Positive ^a^/ Total Samples (Incidence, ^b^ %)	LOD-LOQ ^c^	Mean conc. ^d^ (µg/kg)	Range (µg/kg)	RSD (%)
Littoral region (Mahdia)	5/75 (6.66%)	AFB2	1/5 (20%)	-	0.31	-	-
		AFG2	4/5 (80%)	1	0.33	0.03–0.66	70.28
Continental region (Béja)	12/75 (16%)	AFB1	1/12 (8.33)	-	0.38	-	-
		AFB2	2/12 (16.66)	-	0.30	0.24–0.36	21.20
		AFG2	9/12 (75%)	-	0.37	0.16–0.72	45.40

^a^ Number of positive samples > LOQ, ^b^ incidence of samples > LOQ (% of samples > LOQ), ^c^ number of samples < LOD and >LOQ, ^d^ mean value for samples > LOD.

**Table 10:** Estimated Daily Intake (*EDI*), Hazard Index (*HI*), and (%) of the tolerable daily intake (*TDI*).

Detected Mycotoxins	*EDI* (ng/kg b.w./Day) ^a^	*HI* ^b^	%*TDI* ^c^
**Wheat**			
AFG2	2.35	-	-
OTA	51.6	3.03	303.2
**Milk**			
AFG2	1.5	-	-
AFB1	1.6	-	
AFB2	1.3	-	-

^a^ *EDI*: Estimated Daily Intake; ^b^ *HI*: Hazard Index; ^c^ *TDI*: tolerable daily intake.

**Table 11:** Geographical characteristics of the agro-ecological sampling regions.

Sampling Region	Geographic Position	Altitude (m)	Latitude	Longitude	Bioclimatic Zone
Littoral center region	Center East	6	36807 N	10_22051 E	Semi-Arid Inferior
Continental north region	Northwest	93	36_33004 N	9_26035 W	Sub-Humid

**Table 12:** Sampling details.

Sample		Region	Number of Samples
Durum Wheat	Stored (Summer 2020)	Littoral region Continental region	27 30 **Total: *n* = 57**
	Pre-stored (Summer 2021)	Littoral region Continental region	11 22 **Total: *n* = 33**
Cow Milk		Littoral region Continental region	75 75 **Total: *n* = 150**

## Data Availability

Not applicable.
